# Association between glycemic control and risk of venous thromboembolism in diabetic patients: a nested case–control study

**DOI:** 10.1186/s12933-021-01432-1

**Published:** 2022-01-04

**Authors:** Sarah H. R. Charlier, Christian Meier, Susan S. Jick, Christoph R. Meier, Claudia Becker

**Affiliations:** 1 Basel Pharmacoepidemiology Unit, Division of Clinical Pharmacy and Epidemiology, Department of Pharmaceutical Sciences, University of Basel, Spitalstrasse 26, 4056 Basel, Switzerland; 2 Hospital Pharmacy, University Hospital Basel, Basel, Switzerland; 3 Division of Endocrinology, Diabetes & Metabolism, University Hospital Basel, Basel, Switzerland; 4 Boston Collaborative Drug Surveillance Program, Lexington, USA; 5 School of Public Health, Boston University, Boston University School of Medicine, Lexington, USA

**Keywords:** Diabetes mellitus type 2, Venous thromboembolism, VTE, Glycemic control, HbA1c, Sex, Case–control study

## Abstract

**Background:**

Previous studies suggested an elevated risk of venous thromboembolism (VTE) among patients with type 2 diabetes mellitus (T2DM), with a possible sex difference. The impact of glycemic control on the risk of VTE is unclear. Our objective was to analyze the association between glycemic control and the risk of unprovoked (idiopathic) VTE in men and women with T2DM.

**Methods:**

We conducted a nested case–control analysis (1:4 matching) within a cohort of patients with incident T2DM between 1995 and 2019 using data from the CPRD GOLD. We excluded patients with known risk factors for VTE prior to onset of DM. Cases were T2DM patients with an unprovoked treated VTE. The exposure of interest was glycemic control measured as HbA1c levels. We conducted conditional logistic regression analyses adjusted for several confounders.

**Results:**

We identified 2′653 VTE cases and 10′612 controls (53.1% females). We found no association between the HbA1c level and the risk of VTE in our analyses. However, when the most recent HbA1c value was recorded within 90 days before the index date, women with HbA1c levels > 7.0% had a 36–55% increased relative risk of VTE when compared to women with HbA1c > 6.5–7.0%.

**Conclusions:**

Our study raises the possibility that female T2DM patients with HbA1c levels > 7% may have a slightly higher risk for unprovoked VTE compared to women with HbA1c levels > 6.5–7.0%. This increase may not be causal and may reflect differences in life style or other characteristics. We observed no effect of glycemic control on the risk of VTE in men.

**Supplementary Information:**

The online version contains supplementary material available at 10.1186/s12933-021-01432-1.

## Introduction

Diabetes mellitus (DM) is a chronic disease with a high global prevalence, affecting some 450 million (8.8%) patients worldwide and causing approximately 5 million deaths per year [1]. In the UK, 4.7 million patients (7.0%) had diagnosed or undiagnosed DM in 2019 [2]. Because the majority (90%) of the cases are DM type 2 (T2DM) [2], T2DM and its complications are of great importance for the health system [1]. T2DM is characterized by hyperglycemia due to insulin deficiency and insulin resistance, and it is linked to an increased risk for several cardiovascular diseases [3, 4].

While it has been shown that T2DM patients have a higher risk for arterial thrombosis, the association between T2DM and the risk of venous thromboembolism (VTE) has been studied less. VTE, a medical condition in which a thrombus forms in the venous system, can manifest as deep vein thrombosis (DVT) or as pulmonary embolism (PE), if the thrombus travels to the pulmonary arteries [5–7]. VTE is associated with a high mortality [5, 8]. Its prevention and management is a priority for the NHS, the National Health Service of the UK [9]. Unprovoked VTE [5] occurs at an incidence of 62.1 per 100′000 person years [10]. However, especially at older ages (> 60 years), men have an approximately 20%–25% higher incidence rate of VTE than women [7, 11]. The term unprovoked is used in accordance with the definition provided by the NICE (National Institute for Health and Care Excellence) guideline on PE and DVT, meaning that—similar to the term idiopathic—no recent known major risk factors were present prior to the VTE [5].

Published findings regarding DM as an independent risk factor for VTE are not consistent [6, 12] However, it is well established that VTE occurs more than twice as often in patients with DM than in DM-free individuals [13, 14]. Studies also show that men are at a higher risk for T2DM and VTE than women when both diseases are considered individually, while women are at higher risk of VTE once other comorbidities (such as DM, cardiovascular disease, and atherosclerosis) are involved [12, 15, 16].

Since the degree of hyperglycemia is crucial in the development of DM-related complications [17, 18], the question arises whether there is also an association between hyperglycemia and the risk of VTE.

To date, recent studies assessing the impact of glycemic control on the risk of VTE in male and female patients with DM yielded conflicting results. While some authors found a statistically significant association between the level of glycemic control and the risk of VTE [19, 20], others did not [21]. In a population-based cohort study from Norway, the risk of VTE increased by 5% per one standard deviation increase in HbA1c. However, in this study, there were no HbA1c measurements available at a time point close to the VTE event [21]. None of the published studies analyzed the impact of glycemic control on the risk of VTE stratified by sex. However, the sex of the patient could not only have an impact on the development and progression of the disease itself [15, 22–24], but also on the association of glycemic control and risk of VTE.

A hypothesized pathway for an increased risk of VTE in patients with DM is that hyperglycemia contributes to elevated coagulation factors and impaired fibrinolysis [4, 13, 25]. A single unifying mechanism of DM complications might be hyperglycemia-induced overproduction of superoxide by the mitochondrial electron transport chain, which activates several damaging pathways [26]. The activation of these pathways causes additional intracellular oxidative stress, abnormalities of the gene expression of glomerular cells, hyperglycemia-induced cardiomyocyte dysfunction, and an increase of the enzyme GFAT (glutamine fructose-6 phosphate amidotransferase), resulting in a variety of effects on gene expression and advanced glycation end product formation [26].

The objective of the present study was therefore to analyze the association between glycemic control and the risk of unprovoked VTE in patients with T2DM overall, as well as separately for men and women.

## Methods

### Study design and data source

We conducted a nested case–control analysis within a cohort of patients with incident T2DM between 01. January 1995 and 31. December 2019 in the UK-based primary care Clinical Practice Research Datalink (CPRD) GOLD.

CPRD GOLD contains anonymized medical records of over 11.3 million patients from more than 600 general practices in the UK. It is a governmental, non-profit database; the enrolled patients account for approximately 6.9% of the UK population. Patients within CPRD GOLD are representative of the UK general population with respect to age, sex, and ethnicity [27]. The database was established in 1987 and is a collaborative project between the National Institute for Health Research (NIHR) and the Medicines and Healthcare Regulatory Agency (MHRA). The information in the database comes from participating general practitioners (GPs), who are trained on recording medical information using standard software and coding systems. Medical diagnoses, referrals to specialists and secondary care settings, prescriptions, diagnostic testing, lifestyle information, and demographic data are all part of the recorded information [28]. Many validation studies have been performed that demonstrate the high quality of CPRD GOLD data [28–30]. The validity of the diagnoses of T2DM and VTE has been shown previously [31–33].

### Study population

In order to ensure that we only included incident DM cases in the study population, patients had to have a minimum of 3 years of DM-free history in the database prior to onset. We identified patients based on specific codes for T2DM. We also included patients with an unspecific code for DM (e.g. general code for “diabetes”) if they were older than 30 years at diagnosis and received an oral antidiabetic drug (OAD). Independently of age, if DM patients never received insulin, we classified them as T2DM patients. We used the onset of DM as the study entry date, defined as the date of the first recorded DM code or the date of the first prescription for a DM medication. If the prescription occurred more than 365 days prior to the first recording of a DM diagnosis code, we excluded the patient.

We excluded patients with a diagnosis of cancer (except non-melanoma skin cancer), alcoholism, or HIV at any time in the patient record to avoid substantial bias and confounding.

We excluded patients with a history of VTE (at any time prior to the diagnosis of T2DM), or a code for surgery, immobilization, trauma, paralysis and paresis, or use of HRT or the contraceptive pill within 3 months prior to the index date. We further excluded patients with a code for pregnancy or puerperium within 12 months prior to the index date.

### Case and control definition

We defined cases as patients with a first-time recording of VTE during the study period, who received at least one prescription for an antithrombotic drug within 7 days prior until 90 days after the VTE [5, 34, 35], including vitamin K antagonists, heparins, direct factor Xa inhibitors, direct thrombin inhibitors, fibrinolytic enzymes, or the synthetic penta-saccharide factor Xa inhibitor fondaparinux. The index date for each case was the date of the first recorded VTE. Since we excluded patients with known risk factors for a VTE prior to the outcome, we regard the VTE cases included in this study as having an unprovoked or idiopathic VTE [5].

We used risk set sampling to match each case to 4 controls from the study population, i.e. patients who did not experience a VTE between the onset of DM and the index date of their matched case. We matched controls to cases on age (± 3 years), sex, general practice, index date (same index date as the case, and the control had to be present in the database on the index date), and T2DM duration (± 365 days assessed by counting the days between the study entry date and the index date).

### Exposure definition

The exposure of interest in this study was glycemic control after the onset of DM defined by HbA1c levels. We used the last recorded HbA1c value before the index date for our analyses. We assessed HbA1c levels in 7 categories: ≤ 6.5% (≤ 48 mmol/mol), > 6.5–7.0% (> 48–53 mmol/mol, reference group), > 7.0–7.5% (> 53–58 mmol/mol), > 7.5–8.0% (> 58–64 mmol/mol), > 8.0–9.0% (> 64–75 mmol/mol), > 9.0% (> 75 mmol/mol), and no HbA1c measurement. Results for patients with missing values were presented in a separate category.

### Statistical analysis

We used conditional logistic regression to assess the association between levels of glycemic control (expressed as HbA1c levels) with HbA1c levels of > 6.5–7.0% (> 48–53 mmol/mol) as the reference group and the risk of VTE, expressed as odds ratios (ORs) or adjusted ORs (aORs) with 95% confidence intervals (CI). We also assessed the association between HbA1c level and the risk of VTE according to the patients’ number of GP visits during the study period. Lastly, we conducted analyses in men and women separately.

We adjusted for the following comorbidities and co-medications (recorded at any time in the patient record before the index date) in the final model based on previous clinical knowledge: BMI (categorical variable), smoking status (current, past, non-smokers, and unknown), CVD (including congestive heart failure, ischemic heart disease, myocardial infarction, hypertension, and stroke), osteoarthritis, use of insulin, bisphosphonates, systemic corticosteroids, low-dose acetylsalicylic acid, and current (last prescription within 30 before the index date) or past (last prescription > 30 days prior to index date) use of metformin or sulfonylureas. We additionally tested for effect modification by obesity status (non-obese versus obese, defined as BMI levels < 30 and ≥ 30) of the association between level of HbA1c and risk of VTE.

In sensitivity analyses, we 1) restricted the sample to patients whose last HbA1c measurement was recorded within less than 90 days prior to the index date, 2) analyzed the risk of VTE separately for patients with a previous CVD diagnosis, and 3) conducted separate analyses of the risk of VTE by HbA1c levels for patient groups of different T2DM durations.

We conducted analyses using SAS software version 9.4 (SAS Institute, Inc., Cary, NC, USA).

## Results

**Fig. 1:**
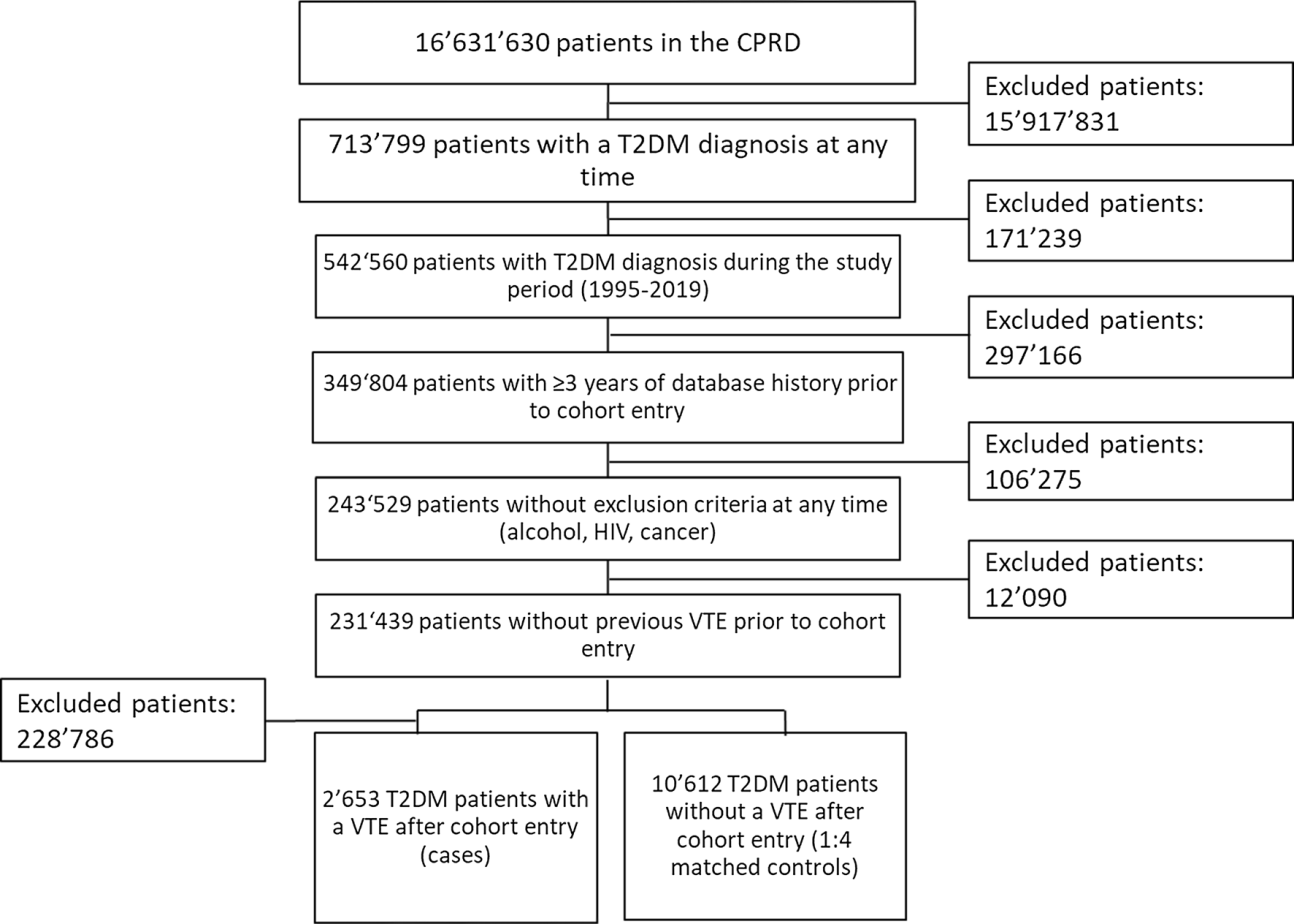
Selection of the study population

Within a cohort of 231′439 patients with incident T2DM who fulfilled all study inclusion and exclusion criteria, we identified 2′653 T2DM patients with an incident VTE diagnosis and 10′612 matched control patients (Fig. 1).

Cases and controls were similar with respect to age and time from most recent HbA1C value to index date. We observed a mean of 12.5 HbA1c measurements per case and 12.3 HbA1c measurements per control during the study period. The median time between the index date and the last HbA1c measurement was 117 days for cases and 116 for controls.

Patients exposed to insulin (aOR 1.63, 95% CI 1.38–1.92) had an increased risk of VTE compared to never-users of insulin, independently of HbA1c levels. However, cases and controls who had at least one prescription for insulin also had longer mean T2DM duration than non-users of these drugs (approximately 4.2 years longer). We found no effect modification by BMI on the association between HbA1c level and the risk of VTE.

Table 1 provides information on the basic characteristics of cases and their matched controls at the index date.

**Table 1:** Characteristics of the included cases and controls

Characteristics	Number of cases (%)	Number of controls (%)	Unadjusted ORs (95% CI)	Adjusted ORs* (95% CI)
Age (years)				
< 60	526 (19.8)	2112 (19.9)	NA	NA
60–69	570 (21.5)	2283 (21.5)	NA	NA
70–79	872 (32.9)	3500 (33.0)	NA	NA
80 +	685 (25.8)	2717 (25.6)	NA	NA
Sex				
Male	1245 (46.9)	4980 (46.9)	NA	NA
Female	1408 (53.1)	5632 (53.1)	NA	NA
BMI (kg/m^2^)				
< 18.5	23 (0.9)	81 (0.8)	1.42 (0.93–2.18)	1.30 (0.82–2.07)
18.5 to < 25.0	340 (12.8)	1779 (16.8)	1 (reference)	1 (reference)
25.0 to < 30.0	770 (29.0)	3705 (34.9)	1.12 (0.99–1.28)	1.15 (1.01–1.31)
30.0 to < 35.0	742 (28.0)	2823 (26.6)	1.47 (1.28–1.68)	1.46 (1.28–1.67)
35.0 to < 40.0	371 (14.0)	1208 (11.4)	1.80 (1.54–2.11)	1.72 (1.47–2.02)
≥ 40.0	343 (12.9)	749 (7.1)	2.82 (2.39–3.34)	2.65 (2.24–3.15)
Unknown	64 (2.4)	267 (2.5)	1.26 (0.96–1.67)	1.30 (0.95–1.79)
Smoking status				
Non-smoker	1001 (37.7)	4301 (40.5)	1 (reference)	1 (reference)
Current smoker	304 (11.5)	1257 (11.9)	1.04 (0.91–1.19)	1.00 (0.87–1.14)
Ex-smoker	1312 (49.5)	4908 (46.3)	1.16 (1.07–1.27)	1.05 (0.96–1.15)
Unknown	36 (1.4)	146 (1.4)	1.04 (0.74–1.47)	1.04 (0.71–1.54)
No. of HbA1c measurements in the medical history before the index date				
1–4	946 (35.7)	4006 (37.8)	1 (reference)	1 (reference)
5–9	1087 (41.0)	4375 (41.2)	1.06 (0.96–1.19)	1.06 (0.95–1.19)
10 or more	365 (13.8)	1343 (12.7)	1.19 (1.02–1.40)	1.06 (0.90–1.26)
No Recording	255 (9.6)	886 (8.4)	1.26 (1.06–1.50)	1.30 (1.08–1.55)
Comorbidities				
Inflammatory bowel disease	107 (4.0)	204 (1.9)	2.17 (1.76–2.66)	1.82 (1.47–2.25)
Chronic renal failure	223 (8.4)	626 (5.9)	1.51 (1.31–1.75)	1.26 (1.08–1.46)
Diabetic retinopathy	835 (31.5)	3090 (29.1)	1.17 (1.07–1.29)	1.20 (1.08–1.32)
Asthma	564 (21.3)	1718 (16.2)	1.41 (1.29–1.55)	1.04 (0.93–1.15)
Congestive heart failure (CHF)	315 (11.9)	712 (6.7)	1.93 (1.71–2.19)	1.53 (1.34–1.76)
Ischemic heart disease (IHD)	690 (26.0)	2189 (20.6)	1.38 (1.26–1.51)	1.21 (1.08–1.35)
Myocardial infarction (MI)	293 (11.0)	996 (9.4)	1.21 (1.07–1.37)	0.93 (0.80–1.07)
Stroke	358 (13.5)	1220 (11.5)	1.21 (1.08–1.35)	1.19 (1.05–1.34)
Arterial hypertension	1697 (60.4)	6866 (64.7)	0.97 (0.89–1.05)	0.93 (0.85–1.01)
Peripheral arterial disease	139 (5.2)	340 (3.2)	1.68 (1.41–2.01)	1.52 (1.26–1.84)
Osteoarthritis	985 (37.1)	3057 (28.8)	1.52 (1.40–1.65)	1.37 (1.25–1.50)
Rheumatoid arthritis	94 (3.5)	226 (2.1)	1.69 (1.37–2.08)	1.29 (1.03–1.62)
Hyperlipidemia	657 (24.8)	2640 (24.9)	0.99 (0.91–1.09)	0.91 (0.83–1.00)
Cardiovascular disease	2033 (76.6)	7916 (74.6)	1.14 (1.03–1.25)	1.05 (0.90–1.24)
Co-medication **				
Insulin	345 (13.0)	820 (7.7)	1.93 (1.67–2.23)	1.63 (1.38–1.92)
Glitazones	367 (13.8)	1227 (11.6)	1.28 (1.12–1.47)	1.09 (0.94–1.26)
Sulfonylurea	1009 (38.0)	3651 (34.4)	1.22 (1.10–1.34)	1.12 (0.99–1.26)
Metformin	1621 (61.1)	6537 (61.6)	0.97 (0.88–1.07)	0.64 (0.41–1.01)
GLP1	81 (3.1)	240 (2.3)	1.44 (1.09–1.90)	0.94 (0.70–1.27)
DPP4	224 (8.4)	926 (8.7)	0.96 (0.81–1.13)	0.84 (0.71–1.00)
SGLT2	43 (1.6)	170 (1.6)	1.01 (0.71–1.46)	0.99 (0.68–1.44)
All oral antidiabetics	1814 (68.4)	7142 (67.3)	1.06 (0.96–1.17)	1.17 (0.95–1.43)
Statins	1941 (73.2)	8077 (76.1)	0.81 (0.72–0.91)	0.74 (0.66–0.84)
Bisphosphonates	340 (12.8)	850 (8.0)	1.79 (1.55–2.06)	1.49 (1.28–1.74)
Contraceptive pill	53 (2.0)	263 (2.5)	0.72 (0.50–1.03)	0.77 (0.53–1.12)
Hormone replacement therapy	392 (14.8)	1626 (15.3)	0.94 (0.82–1.09)	0.89 (0.77–1.03)
Corticosteroids (systemic)	975 (36.8)	2612 (24.6)	1.84 (1.67–2.02)	1.55 (1.40–1.71)
Coronary vasodilators	888 (33.5)	2797 (26.4)	1.43 (1.30–1.57)	1.22 (1.07–1.39)
Low dose acetylsalicylic acid	1556 (58.7)	5991 (56.5)	1.11 (1.01–1.22)	0.95 (0.86–1.06)
Loop diuretics	1152 (43.4)	2875 (27.1)	2.24 (2.04–2.46)	1.69 (1.52–1.88)
All diuretics	1854 (69.9)	6323 (59.6)	1.73 (1.56–1.91)	1.52 (1.36–1.70)

*Adjusted for BMI (categorical), smoking (categorical), CHF, IHD, MI, stroke, hypertension, osteoarthritis, and use of insulin, bisphosphonate, systemic corticosteroids, low-dose acetylsalicylic acid, current and past use of metformin, and current and past use of sulfonylureas

**Use of other medication possible

We found no elevated relative risk for VTE in patients with the last HbA1c measurement > 7.0% (> 53 mmol/mol) compared to the reference group of patients with HbA1c levels > 6.5–7.0% (> 48–53 mmol/mol). The ORs for the various HbA1c categories are displayed in Table 2. There was no consistent linear increase in the risk of developing VTE with increasing HbA1c levels. Patients with missing HbA1c measurements had the highest risk of VTE (aOR 1.56, 95% CI 1.29–1.88) when compared to patients with last HbA1c measurements of > 6.5–7.0% (> 48–53 mmol/mol) before the index date. Around one third of the cases with no HbA1c measurements (8.4% in total) had little GP contact (0–14 GP visits: 34.7% (n = 77), 15–29 visits: 18.5% (n = 41), and 30 + visits: 46.9% (n = 104). We provide a separate table summarizing the characteristics of those patients without any HbA1c measurements as a Supplement.

**Table 2:** Risk of VTE by HbA1c level

HbA1c-level	Number of cases (%)	Number of controls (%)	Unadjusted ORs (95% CI)	Adjusted ORs* (95% CI)
HbA1c-Values (last measurement before the index date) and risk of VTE				
≤ 6.5% (≤ 48 mmol/mol)	843 (31.8)	3809 (35.9)	0.98 (0.87–1.10)	0.98 (0.87–1.10)
> 6.5–7.0% (> 48– 53 mmol/mol)	441 (16.6)	1963 (18.5)	1 (reference)	1 (reference)
> 7.0–7.5% (> 53– 58 mmol/mol)	397 (15.0)	1522 (14.3)	1.17 (1.02–1.34)	1.13 (0.98–1.30)
> 7.5–8.0% (> 58– 64 mmol/mol)	208 (7.8)	839 (7.9)	1.13 (0.95–1.33)	1.09 (0.92–1.29)
> 8.0–9.0% (> 64– 75 mmol/mol)	253 (9.5)	834 (7.9)	1.38 (1.18–1.61)	1.30 (1.10–1.52)
> 9.0% (> 75 mmol/mol))	289 (10.9)	929 (8.8)	1.45 (1.24–1.69)	1.18 (1.00–1.40)
No Recording	222 (8.4)	716 (6.8)	1.54 (1.28–1.85)	1.56 (1.29–1.88)
HbA1c-Values (last measurement before the index date) and risk of VTE in women				
≤ 6.5% (≤ 48 mmol/mol)	468 (33.2)	2106 (37.4)	1.04 (0.89–1.22)	1.05 (0.89–1.23)
> 6.5–7.0% (> 48– 53 mmol/mol)	220 (15.6)	1035 (18.4)	1 (reference)	1 (reference)
> 7.0–7.5% (> 53– 58 mmol/mol)	211 (15.0)	809 (14.4)	1.24 (1.03–1.50)	1.16 (0.95–1.41)
> 7.5–8.0% (> 58– 64 mmol/mol)	102 (7.2)	422 (7.5)	1.16 (0.92–1.47)	1.15 (0.90–1.48)
> 8.0–9.0% (> 64– 75 mmol/mol)	122 (8.7)	411 (7.3)	1.43 (1.15–1.79)	1.29 (1.02–1.63)
> 9.0% (> 75 mmol/mol))	154 (10.9)	456 (8.10)	1.68 (1.36–2.09)	1.36 (1.07–1.72)
No Recording	131 (9.3)	393 (7.0)	1.81 (1.42–2.31)	1.87 (1.46–2.40)
HbA1c-Values (last measurement before the index date) and risk of VTE in men				
≤ 6.5% (≤ 48 mmol/mol)	375 (30.1)	1703 (34.2)	0.92 (0.78–1.09)	0.91 (0.77–1.08)
> 6.5–7.0% (> 48– 53 mmol/mol)	221 (17.8)	928 (18.6)	1 (reference)	1 (reference)
> 7.0–7.5% (> 53– 58 mmol/mol)	186 (14.9)	713 (14.3)	1.10 (0.91–1.34)	1.10 (0.90–1.34)
> 7.5–8.0% (> 58–64 mmol/mol)	106 (8.5)	417 (8.4)	1.09 (0.86–1.37)	1.03 (0.81–1.30)
> 8.0–9.0% (> 64–75 mmol/mol)	131 (10.5)	423 (8.5)	1.33 (1.07–1.65)	1.29 (1.03–1.62)
> 9.0% (> 75 mmol/mol))	135 (10.8)	473 (9.5)	1.24 (0.99–1.55)	1.03 (0.81–1.30)
No Recording	91 (7.3)	323 (6.5)	1.26 (0.95–1.66)	1.24 (0.93–1.66)

*Adjusted for BMI (categorical), smoking (categorical), CHF, IHD, MI, stroke, hypertension, osteoarthritis, and use of insulin, bisphosphonate, systemic corticosteroids, low-dose acetylsalicylic acid, current and past use of metformin, and current and past use of sulfonylureas

When we stratified our analyses by sex (Table 2), we observed a slightly higher risk of VTE in women with HbA1c levels > 8.0% (> 64 mmol/mol) compared to the reference group of women with HbA1c levels > 6.5–7.0% (> 48–53 mmol/mol). There was no association between HbA1c levels and risk of VTE in men.

Among patients with preexisting CVD (Table 3), individuals with HbA1c levels > 7.0% (> 53 mmol/mol) had a similar risk of VTE compared to patients with HbA1c levels between > 6.5–7.0% (> 48–53 mmol/mol). There was a slightly higher risk of VTE with increased HbA1c levels in women with CVD, but not in men.

**Table 3:** Risk of VTE according to HbA1c in patients with CVD

Characteristics	Number of cases (%)	Number of controls (%)	Unadjusted ORs (95% CI)	Adjusted ORs* (95% CI)
CVD and risk of VTE				
≤ 6.5%	670 (33.0)	2998 (37.9)	0.99 (0.87–1.13)	1.01 (0.89–1.15)
> 6.5–7.0%	352 (17.3)	1524 (19.3)	1 (reference)	1 (reference)
> 7.0–7.5%	307 (15.1)	1142 (14.4)	1.19 (1.02–1.39)	1.14 (0.97–1.34)
> 7.5–8.0%	163 (8.0)	610 (7.7)	1.18 (0.98–1.43)	1.13 (0.93–1.38)
> 8.0–9.0%	193 (9.5)	568 (7.2)	1.49 (1.24–1.78)	1.39 (1.16–1.68)
> 9.0%	201 (9.9)	624 (7.9)	1.47 (1.22–1.77)	1.16 (0.95–1.42)
No Recording	147 (7.2)	450 (5.7)	1.55 (1.25–1.94)	1.56 (1.24–1.96)
HbA1c-Values (last measurement before the index date) and risk of VTE in women				
≤ 6.5%	377 (34.4)	1689 (39.2)	1.04 (0.87–1.23)	1.07 (0.90–1.28)
> 6.5–7.0%	177 (16.2)	818 (19.0)	1 (reference)	1 (reference)
> 7.0–7.5%	166 (15.2)	618 (14.4)	1.29 (1.04–1.59)	1.21 (0.96–1.51)
> 7.5–8.0%	87 (7.9)	317 (7.4)	1.30 (1.00–1.69)	1.26 (0.95–1.66)
> 8.0–9.0%	93 (8.5)	285 (6.6)	1.39 (1.08–1.80)	1.30 (0.99–1.70)
> 9.0%	107 (9.8)	318 (7.4)	1.64 (1.28–2.12)	1.27 (0.97–1.68)
No Recording	89 (8.1)	260 (6.0)	1.80 (1.35–2.41)	1.83 (1.35–2.46)
HbA1c-Values (last measurement before the index date) and risk of VTE in men				
≤ 6.5%	293 (31.3)	1309 (36.3)	0.94 (0.78–1.14)	0.94 (0.78–1.13)
> 6.5–7.0%	175 (18.7)	706 (19.6)	1 (reference)	1 (reference)
> 7.0–7.5%	141 (15.1)	524 (14.5)	1.09 (0.87–1.37)	1.06 (0.84–1.34)
> 7.5–8.0%	76 (8.1)	293 (8.1)	1.07 (0.82–1.41)	0.98 (0.74–1.31)
> 8.0–9.0%	100 (10.7)	283 (7.8)	1.59 (1.23–2.05)	1.50 (1.15–1.96)
> 9.0%	94 (10.0)	306 (8.5)	1.30 (0.99–1.72)	1.06 (0.79–1.41)
No Recording	58 (6.2)	190 (5.3)	1.26 (0.90–1.78)	1.26 (0.88–1.81)

*Adjusted for BMI (categorical), smoking (categorical), CHF, IHD, MI, stroke, hypertension, osteoarthritis, and use of insulin, bisphosphonate, systemic corticosteroids, low-dose acetylsalicylic acid, current and past use of metformin, and current and past use of sulfonylureas

Also in an analysis restricted to patients with a last HbA1c measurement within 90 days prior to the index date (Table 4), we only found a slight association between HbA1c levels > 7.0% (> 53 mmol/mol) and risk of VTE in women. The risk of VTE among women with a 90-day HbA1c level above 7.0% (> 53 mmol/mol) increased around 36–55% as compared to those with HbA1c levels > 6.5–7.0% (> 48–53 mmol/mol).

**Table 4:** Risk of VTE according to HbA1c levels measured within 90 days prior to the index date (i.d.)

	Number of cases (%)	Number of controls (%)	Unadjusted ORs (95% CI)	Adjusted ORs* (95% CI)
HbA1c level < 90 days prior to i.d. overall				
≤ 6.5%	278 (22.9)	1345 (28.6)	1.00 (0.81–1.23)	1.04 (0.84–1.30)
> 6.5–7.0%	161 (13.3)	789 (16.8)	1 (reference)	1 (reference)
> 7.0–7.5%	172 (14.2)	616 (13.1)	1.40 (1.10–1.78)	1.44 (1.11–1.87)
> 7.5–8.0%	108 (8.9)	380 (8.1)	1.43 (1.10–1.88)	1.46 (1.09–1.94)
> 8.0–9.0%	132 (10.9)	394 (8.4)	1.69 (1.30–2.19)	1.64 (1.23–2.17)
> 9.0%	140 (11.5)	460 (9.8)	1.50 (1.17–1.92)	1.32 (1.01–1.73)
No Recording**	222 (18.3)	716 (15.2)	1.75 (1.36–2.23)	1.78 (1.38–2.31)
HbA1c level < 90 days prior to i.d. in women				
≤ 6.5%	155 (11.0)	746 (13.3)	0.99 (0.74–1.34)	1.03 (0.75–1.40)
> 6.5–7.0%	82 (5.8)	417 (7.4)	1 (reference)	1 (reference)
> 7.0–7.5%	99 (7.0)	335 (6.0)	1.47 (1.05–2.06)	1.55 (1.08–2.24)
> 7.5–8.0%	51 (3.6)	192 (3.4)	1.36 (0.92–2.01)	1.43 (0.93–2.18)
> 8.0–9.0%	66 (4.7)	208 (3.7)	1.50 (1.04–2.15)	1.36 (0.91–2.03)
> 9.0%	79 (5.6)	239 (4.2)	1.61 (1.13–2.29)	1.47 (0.99–2.17)
No Recording**	876 (62.2)	3495 (62.1)	1.94 (1.39–2.71)	1.98 (1.40–2.81)
HbA1c level < 90 days prior to i.d. in men				
≤ 6.5%	125 (10.0)	614 (12.3)	1.00 (0.75–1.34)	1.04 (0.76–1.43)
> 6.5–7.0%	81 (6.5)	379 (7.6)	1 (reference)	1 (reference)
> 7.0–7.5%	76 (6.1)	292 (5.9)	1.32 (0.93–1.86)	1.35 (0.91–1.99)
> 7.5–8.0%	58 (4.7)	191 (3.8)	1.50 (1.04–2.17)	1.48 (0.99–2.22)
> 8.0–9.0%	67 (5.4)	186 (3.7)	1.93 (1.33–2.80)	1.94 (1.28–2.96)
> 9.0%	62 (5.0)	226 (4.5)	1.37 (0.96–1.95)	1.23 (0.83–1.82)
No Recording**	776 (62.3)	3092 (62.1)	1.50 (1.04–2.17)	1.56 (1.04–2.34)

*Adjusted for BMI (categorical), smoking (categorical), CHF, IHD, MI, stroke, hypertension, osteoarthritis, and use of insulin, bisphosphonate, systemic corticosteroids, low-dose acetylsalicylic acid, current and past use of metformin, and current and past use of sulfonylureas

**Included are patients with missing HbA1c measurements as well as those with a last HbA1c level recorded > 90 days prior to the index date

We found no association between the risk of VTE by HbA1c level in the group of patients with a T2DM duration of more than 5 years (Table 5). However, among patients with shorter T2DM duration (0–5 years), those with HbA1c levels > 7.0% (> 53 mmol/mol) had slightly higher aORs for VTE when compared to T2DM patients with HbA1c levels of > 6.5–7.0% (HbA1c > 7.0–7.5%: aOR 1.20, 95% CI 0.97–1.49; HbA1c > 7.5–8.0%: aOR 1.29, 95% CI 1.00–1.67; HbA1c > 8.0–9.0%: aOR 1.44, 95% CI 1.13–1.83; HbA1c > 9.0%: aOR 1.39, 95% CI 1.07–1.79).

**Table 5:** Risk of VTE in patients with different T2DM durations

	Number of cases (%)	Number of controls (%)	Unadjusted ORs (95% CI)	Adjusted ORs* (95% CI)
Last HbA1c: 0–5 y since T2DM diagnosis				
≤ 6.5% (≤ 48 mmol/mol)	446 (33.7)	2042 (38.6)	1.02 (0.86–1.20)	1.06 (0.89–1.25)
> 6.5–7.0% (> 48–53 mmol/mol)	210 (15.9)	990 (18.7)	1 (reference)	1 (reference)
> 7.0–7.5% (> 53–58 mmol/mol)	172 (13.0)	651 (12.3)	1.24 (1.01–1.51)	1.20 (0.97–1.49)
> 7.5–8.0% (> 58–64 mmol/mol)	86 (6.5)	326 (6.2)	1.30 (1.01–1.68)	1.29 (1.00–1.67)
> 8.0–9.0% (> 64–75 mmol/mol)	104 (7.9)	336 (6.4)	1.51 (1.20–1.91)	1.44 (1.13–1.83)
> 9.0% (> 75 mmol/mol))	114 (8.6)	348 (6.6)	1.61 (1.27–2.03)	1.39 (1.07–1.79)
No Recording	191 (14.4)	596 (11.3)	1.75 (1.40–2.19)	1.78 (1.41–2.24)
Last HbA1c: 5–10 y since T2DM diagnosis				
≤ 6.5% (≤ 48 mmol/mol)	260 (32.1)	1168 (35.8)	0.84 (0.69–1.02)	0.83 (0.68–1.01)
> 6.5–7.0% (> 48–53 mmol/mol)	161 (19.9)	618 (19.0)	1 (reference)	1 (reference)
> 7.0–7.5% (> 53–58 mmol/mol)	138 (17.1)	525 (16.1)	1.00 (0.80–1.27)	0.97 (0.76–1.23)
> 7.5–8.0% (> 58–64 mmol/mol)	58 (7.2)	284 (8.7)	0.78 (0.58–1.05)	0.73 (0.53–1.01)
> 8.0–9.0% (> 64–75 mmol/mol)	80 (9.9)	270 (8.3)	1.19 (0.90–1.57)	1.11 (0.83–1.48)
> 9.0% (> 75 mmol/mol))	85 (10.5)	306 (9.4)	1.07 (0.81–1.40)	0.87 (0.65–1.16)
No Recording	27 (3.3)	88 (2.7)	1.24 (0.81–1.91)	1.46 (0.93–2.31)
Last HbA1c: > 10 y since T2DM diagnosis				
≤ 6.5% (≤ 48 mmol/mol)	137 (26.3)	599 (29.0)	1.16 (0.87–1.55)	1.10 (0.81–1.48)
> 6.5–7.0% (> 48–53 mmol/mol)	70 (13.4)	355 (17.2)	1 (reference)	1 (reference)
> 7.0–7.5% (> 53–58 mmol/mol)	87 (16.7)	346 (16.8)	1.29 (0.95–1.76)	1.20 (0.87–1.64)
> 7.5–8.0% (> 58–64 mmol/mol)	64 (12.3)	229 (11.1)	1.42 (1.00–2.02)	1.36 (0.94–1.96)
> 8.0–9.0% (> 64–75 mmol/mol)	69 (13.2)	228 (11.1)	1.53 (1.10–2.14)	1.40 (0.98–1.99)
> 9.0% (> 75 mmol/mol))	90 (17.3)	275 (13.3)	1.74 (1.26–2.40)	1.33 (0.92–1.91)
No Recording	4 (0.8)	32 (1.6)	0.56 (0.23–1.37)	0.58 (0.22–1.52)

*Adjusted for BMI (categorical), smoking (categorical), CHF, IHD, MI, stroke, hypertension, osteoarthritis, and use of insulin, bisphosphonate, systemic corticosteroids, low-dose acetylsalicylic acid, current and past use of metformin, and current and past use of sulfonylureas

## Discussion

In this large case–control study based on primary care data from the UK, patients with HbA1c > 7.0% (> 53 mmol/mol) did not have an increased risk of unprovoked VTE compared to patients with HbA1c > 6.5–7.0% (> 48–53 mmol/mol). In the subset of female patients, we found a suggestion of a slightly increased risk of VTE in women with HbA1c > 8.0% (for example HbA1c > 8.0–9.0%: aOR 1.29, 95% CI 1.02–1.63) when compared to those with HbA1c > 6.5–7.0% (> 48–53 mmol/mol). This increase was slightly more pronounced if we only considered patients with HbA1c measurements taken within 90 days prior to the index date. Overall, however, the association in women was weak, and there was no trend of increasing risk of VTE in association with increasing HbA1c values. We did not observe an increased risk of VTE in men at any level of glycemic control.

The weak association between elevated HbA1c levels and risk of VTE in women, but not in men, may be explained by the fact that pre-diabetic and diabetic women are more affected by chronically elevated cardiovascular risk factors, and their health declines faster when compared to men. [22, 36, 37]. Since T2DM is a disease with uncertain onset, which can remain undiagnosed for many years, this difference in risk factor levels between men and women is relevant. Several studies, including a comprehensive meta-analysis, suggest that the presence of diabetes eliminates the biological female advantage that is often used to explain the lower absolute rates of coronary heart disease (CHD) and stroke in women compared to men [22, 38, 39]. The authors of this meta-analysis estimate that the relative risk for CHD is 44% greater in women with diabetes than in similarly affected men [39]. In general, our study population included more women than men, even though men are more often affected by T2DM and by VTE, when the diseases are observed independently of each other. The T2DM cohort for our study also included more men than women prior to the identification of the VTE cases (51.1% vs 48.9%). Several studies provide an explanation for this imbalance in the rates of affected females and males by showing that adverse changes in metabolic and vascular risk factor profiles are greater in women than in men. These changes occur in diabetic individuals as well as earlier in pre-diabetic individuals [15, 22–24].

Patients with CVD and HbA1c levels > 7.0% (> 53 mmol/mol) did not have an increased risk of VTE when compared to those with HbA1c levels between > 6.5–7.0% (> 48–53 mmol/mol), though women with CVD and HbA1c level > 7% (> 53 mmol/mol) had a slightly elevated risk for VTE, while men with CVD did not. This result emphasizes the general importance of proper glycemic control in women suffering from both, CVD and T2DM.

In our study, patients with no recorded HbA1c measurements had a higher risk of VTE compared to patients with HbA1c > 6.5–7.0% (> 48–53 mmol/mol) throughout our analyses. This could be a proxy for a lack of patient-doctor interaction and poor treatment adherence, which could lead not only to a higher risk for VTE (as suggested in this study), but potentially to other complications caused by improper management of T2DM. This assumption is reinforced by the results shown in Additional file 1: Supplementary Table 1, where patients without HbA1c measurements had much lower numbers for diagnosis of comorbidities, as well as for a corresponding prescription, when compared to patients who had at least 1 HbA1c measurement.

The present findings should be interpreted within the context of the strengths and limitations of an observational study. A delayed diagnosis of T2DM may have led to the inclusion of some prevalent (instead of incident) T2DM cases in our cohort. Additionally, the UK Prospective Diabetes study found that a high prevalence of DM tissue damage was already present by the time the DM diagnosis was made, which is an indication of pre-existing DM [40]. Therefore, we may have underestimated the time until VTE events (after the recorded DM diagnosis) in our study population, which could have potentially affected our matching on DM duration. However, this misclassification is unlikely to have been differential by HbA1c level, and we do not expect that it had a major influence on our findings.

Though VTE events are well recorded and have previously been validated in the CPRD (positive predictive value 88.2% [82.3–92.6%] for VTE) [33], it is possible that we missed some unrecorded VTEs. This possible misclassification would likely be non-differential and would not materially change the results.

We considered BMI, previously diagnosed CVD, use of statins, as well as well as other factors that may increase the risk of VTE (see Table 1) in our analyses. However, we were not in the position to include data on diet, waist circumference, or physical activity, since this data is not available in the CPRD.

The strengths of our study include the large study sample and the observational nested case–control design within a cohort of patients with newly diagnosed T2DM. Our data come from a well validated primary care database that contains prospectively and routinely collected data, which avoids recall bias. Even though we only used the last HbA1c measurement before the index date, HbA1c measurements are regularly performed in the diabetic population, and median time between the index date and the last HbA1c measurement was short. This shows that the recorded HbA1c measurements provide a reliable and timely source for our analyses on the effect of glycemic control on the risk of VTE.

Our study population included a high proportion of patients with T2DM with HbA1c ≤ 7% (> 53 mmol/mol) who may have been healthier than the T2DM populations analyzed in other studies. Nevertheless, our population consisted of over 13′000 patients with T2DM, many of whom had HbA1c levels > 7% (> 53 mmol/mol). Therefore, we expect our results to be generalizable to those of other populations with T2DM and HbA1c levels > 7% (> 53 mmol/mol).

In conclusion, our study provides evidence that HbA1c levels > 7% (> 53 mmol/mol) are not associated with a materially increased risk for unprovoked VTE overall. There was a suggestion of a slightly increased VTE risk in women, which may be real or may reflect differences in lifestyle or other patient characteristics.

## Conclusion

Our study raises the possibility that female T2DM patients with HbA1c levels > 7% may have a slightly higher risk for unprovoked VTE compared to women with HbA1c level > 6.5–7.0%. This increase may not be causal and may reflect differences in life style or other characteristics. We observed no effect of glycemic control on the risk of VTE in men.

## Supplementary Information

**Additional file 1. Table S1.** Characteristics of the included cases and controls without HbA1c-measurement.

## Data Availability

The data that support the findings of this study are available from the CPRD but restrictions apply to the availability of these data, which were used under license for the current study, and so are not publicly available. Data are however available from the authors upon reasonable request and with permission of CPRD.

## Abbreviations

aOR: Adjusted Odds Ratio
BMI: Body Mass Index
CHD: Coronary Heart Disease
CHF: Congestive Heart Failure
CI: Confidence interval
CPRD: Clinical Practice Research Datalink
CVD: Cardiovascular disease
DM: Diabetes mellitus
DVT: Deep vein thrombosis
GFAT: Glutamine Fructose-6 Phosphate Amidotransferase
GP: General practitioner
HbA1c: Glycated hemoglobin A1c
HRT: Hormone replacement therapy
i.d.: Index date
IHD: Ischemic heart disease
ISAC: Independent Scientific Advisory Committee
MHRA: Medicines and Healthcare Products Regulatory Agency
MI: Myocardial infarction
NHS: National Health Services
NIHR: National Institute For Health Research
OAD: Oral antidiabetic drug
OR: Odds ratio
PE: Pulmonary embolism
UK: United Kingdom
T1DM: Type 1 diabetes mellitus
T2DM: Type 2 diabetes mellitus
VTE: Venous thromboembolism
