# Supplementary material for: Association between glycemic control and risk of venous thromboembolism in diabetic patients: a nested case–control study
Source: Cardiovasc Diabetol. 2022 Jan 4;21:2. doi: 10.1186/s12933-021-01432-1 (PMC8729078; doi:10.1186/s12933-021-01432-1)
Supplement: Supplementary file 1 — Additional file 1. Table S1. Characteristics of the included cases and controls without HbA1c-measurement. [file 12933_2021_1432_MOESM1_ESM.docx]

**Additional file 1: Supplementary Table S1:** Characteristics of the included cases and controls without HbA1c-measurement

| **Characteristics** | **Number of cases (%)** | **Number of controls (%)** | | **Unadjusted ORs**  **(95% CI)** | **Adjusted ORs***  **(95% CI)** |
| --- | --- | --- | --- | --- | --- |
| Age (years) | | | | | |
| <60 | 29 (27.9) | 90 (24.7) | | NA | NA |
| 60-69 | 24 (23.1) | 85 (23.4) | | NA | NA |
| 70-79 | 28 (26.9) | 98 (26.9) | | NA | NA |
| 80+ | 23 (22.1) | 91 (25.0) | | NA | NA |
|  | | | | | |
| Male | 63 (60.6) | 197 (54.1) | | NA | NA |
| Female | 41 (39.4) | 167 (45.9) | | NA | NA |
| BMI (kg/m^2^) | | | | | |
| <18.5 | 3 (2.9) | 4 (1.1) | | -- | -- |
| 18.5 to <25.0 | 15 (14.4) | 87 (23.9) | | 1 (reference) | 1 (reference) |
| 25.0 to <30.0 | 30 (28.9) | 103 (28.3) | | 0.82 (0.34-1.96) | 0.35 (0.08-1.49) |
| 30.0 to <35.0 | 15 (14.4) | 66 (18.1) | | 0.98 (0.36-2.69) | 0.16 (0.02-1.02) |
| 35.0 to <40.0 | 14 (13.5) | 27 (7.4) | | 1.85 (0.36-2.69) | 1.35 (0.26-6.91) |
| ≥40.0 | 11 (10.6) | 12 (3.3) | | 4.34 (0.87-22.03) | 3.54 (0.54-23.43) |
| Unknown | 16 (15.4) | 65 (17.9) | | 1.49 (0.43-5.12) | 0.24 (0.03-1.74) |
| Smoking status | | | | | |
| Non-smoker | 44 (42.3) | 159 (43.7) | | 1 (reference) | 1 (reference) |
| Current smoker | 16 (15.4) | 47 (12.9) | | 0.97 (0.42-2.24) | 0.86 (0.25-2.94) |
| Ex-smoker | 33 (31.7) | 105 (28.9) | | 1.03 (0.54-1.95) | 0.85 (0.19-3.78) |
| Unknown | 11 (10.6) | 53 (14.6) | | 1.74 (0.70-4.37) | 1.18 (0.13-10.56) |
| Comorbidities | | | | | |
| Inflammatory bowel disease | 3 (2.9) | | 10 (2.8) | -- | -- |
| Chronic renal failure | 5 (4.8) | | 7 (1.9) | -- | -- |
| Diabetic retinopathy | 7 (6.7) | | 16 (4.4) | 1.00 (0.25-4.00) | 0.60 (0.08-4.62) |
| Asthma | 16 (15.4) | | 51 (14.0) | 2.00 (0.67-5.99) | 1.11 (0.20-6.05) |
| Congestive heart failure (CHF) | 12 (11.5) | | 19 (5.2) | 1.56 (0.53-4.63) | 0.50 (0.11-2.20) |
| Ischemic heart disease (IHD) | 19 (18.3) | | 66 (18.1) | 3.15 (0.26-7.88) | 74.78 (1.64-3416.0) |
| Myocardial infarction (MI) | 9 (8.7) | | 28 (7.7) | 1.86 (0.62-5.56) | 0.10 (0.01-1.40) |
| Stroke | 12 (11.5) | | 33 (9.1) | 0.93 (0.35-2.49) | 4.32 (0.35-53.91) |
| Arterial hypertension | 45 (43.3) | | 172 (47.3) | 1.95 (1.01-3.77) | 3.14 (0.95-10.44) |
| Peripheral arterial disease | 3 (2.9) | | 7 (1.9) | -- | -- |
| Osteoarthritis | 31 (29.8) | | 91 (25.0) | 1.23 (0.64-2.35) | 3.01 (1.26-7.19) |
| Rheumatoid arthritis | 4 (3.9) | | 6 (1.7) | 3.24 (0.48-21.86) | 0.91 (0.07-11.08) |
| Hyperlipidemia | 16 (15.4) | | 45 (12.4) | 3.31 (0.94-11.72) | 4.52 (0.24-86.34) |
| Cardiovascular disease | 61 (58.7) | | 219 (60.2) | 1.87 (0.95-3.72) | 0.09 (0.01-0.94) |
| Co-medication ** | | | | | |
| Insulin | 7 (6.7) | | 9 (2.5) | 3.00 (0.31-28.84) | 18.91 (0.69-516.8) |
| Glitazones | 0 (0.0) | | 0 (0.0) | -- | -- |
| Sulfonylurea | 24 (23.1) | | 61 (16.8) | 1.64 (0.61-4.40) | 2.48 (0.41-14.94) |
| Metformin | 16 (15.4) | | 64 (17.6) | 0.80 (0.29-2.23) | 0.19 (0.03-1.47) |
| GLP1 | 0 (0.0) | | 0 (0.0) | -- | -- |
| DPP4 | 99 (7.5) | | 470 (8.9) | 0.90 (0.69-1.17) | 0.74 (0.55-0.98) |
| SGLT2 | 0 (0.0) | | 0 (0.0) | -- | -- |
| All oral antidiabetics | 32 (30.8) | | 105 (28.9) | 1.16 (0.50-2.69) | 0.04 (0.00-9.41) |
| Statins | 28 (26.9) | | 104 (28.6) | 2.14 (0.62-7.41) | 6.38 (0.50-81.58) |
| Bisphosphonates | 4 (3.9) | | 24 (6.6) | 0.25 (0.03-2.24) | 0.06 (0.00-1.79) |
| Contraceptive pill | 1 (1.0) | | 11 (3.0) | -- | -- |
| Hormone replacement therapy | 10 (9.6) | | 38 (10.4) | 0.15 (0.02-1.21) | 0.05 (0.00-1.40) |
| Corticosteroids (systemic) | 29 (27.9) | | 60 (16.5) | 2.56 (0.86-7.60) | 8.52 (0.88-82.95) |
| Coronary vasodilators | 21 (20.2) | | 62 (17.0) | 1.26 (0.45-3.56) | -- |
| Low dose acetylsalicylic acid | 36 (34.6) | | 104 (28.6) | 1.53 (0.65-3.59) | 0.38 (0.04-3.49) |
| Loop diuretics | 26 (25.0) | | 69 (19.0) | 0.77 (0.23-2.64) | 0.21 (0.02-1.88) |
| All diuretics | 61 (58.7) | | 168 (46.2) | 2.30 (0.81-6.53) | 1.66 (0.29-9.61) |

* Adjusted for BMI (categorical), smoking (categorical), CHF, IHD, MI, stroke, hypertension, osteoarthritis, and use of insulin, bisphosphonate, systemic corticosteroids, low-dose acetylsalicylic acid, current and past use of metformin, and current and past use of sulfonylureas

** Use of other medication possible
